# Human *Streptococcus agalactiae* strains in aquatic mammals and fish

**DOI:** 10.1186/1471-2180-13-41

**Published:** 2013-02-18

**Authors:** Christian MJ Delannoy, Margaret Crumlish, Michael C Fontaine, Jolinda Pollock, Geoff Foster, Mark P Dagleish, James F Turnbull, Ruth N Zadoks

**Affiliations:** 1 Institute of Aquaculture, School of Natural Sciences, University of Stirling, Stirling, UK; 2 Moredun Research Institute, Pentlands Science Park, Bush Loan, Penicuik, UK; 3 SAC Consulting Veterinary Services, Drummondhill, Stratherrick Road, Inverness, UK; 4 Current address: SRUC (formally SAC), The Roslin Institute, The University of Edinburgh, Easter Bush, Midlothian, UK; 5 Current address: Institute of Biodiversity, Animal Health and Comparative Medicine, College of Medical, Veterinary and Life Sciences, University of Glasgow, Scotland, UK

**Keywords:** *Streptococcus agalactiae*, Fish, Sea mammal, Aquatic epidemiology, Molecular epidemiology, MLST, PFGE

## Abstract

**Background:**

In humans, *Streptococcus agalactiae* or group B streptococcus (GBS) is a frequent coloniser of the rectovaginal tract, a major cause of neonatal infectious disease and an emerging cause of disease in non-pregnant adults. In addition, *Streptococcus agalactiae* causes invasive disease in fish, compromising food security and posing a zoonotic hazard. We studied the molecular epidemiology of *S. agalactiae* in fish and other aquatic species to assess potential for pathogen transmission between aquatic species and humans.

**Methods:**

Isolates from fish (n = 26), seals (n = 6), a dolphin and a frog were characterized by pulsed-field gel electrophoresis, multilocus sequence typing and standardized 3-set genotyping, i.e. molecular serotyping and profiling of surface protein genes and mobile genetic elements.

**Results:**

Four subpopulations of *S. agalactiae* were identified among aquatic isolates. Sequence type (ST) 283 serotype III-4 and its novel single locus variant ST491 were detected in fish from Southeast Asia and shared a 3-set genotype identical to that of an emerging ST283 clone associated with invasive disease of adult humans in Asia. The human pathogenic strain ST7 serotype Ia was also detected in fish from Asia. ST23 serotype Ia, a subpopulation that is normally associated with human carriage, was found in all grey seals, suggesting that human effluent may contribute to microbial pollution of surface water and exposure of sea mammals to human pathogens. The final subpopulation consisted of non-haemolytic ST260 and ST261 serotype Ib isolates, which belong to a fish-associated clonal complex that has never been reported from humans.

**Conclusions:**

The apparent association of the four subpopulations of *S. agalactiae* with specific groups of host species suggests that some strains of aquatic *S. agalactiae* may present a zoonotic or anthroponotic hazard. Furthermore, it provides a rational framework for exploration of pathogenesis and host-associated genome content of *S. agalactiae* strains.

## Background

*Streptococcus agalactiae* or group B streptococcus (GBS) is the major cause of invasive neonatal infections in industrialized countries [1,2]. Early and late onset disease in infants of 1 to 6 or at least 7 days old, respectively, are characterized by sepsis and meningitis. Early onset disease usually results from mother-to-child transmission and can be prevented through intrapartum chemoprophylaxis. The routine use of screening protocols and intrapartum chemoprophylaxis has led to decrease in the incidence of early onset disease, whereas the incidence of late onset disease is not affected [1,2]. *Streptococcus agalactiae* also causes a considerable burden of disease in adults, with case fatality rates approximating 15% in countries in North America, Asia and Europe [2-4]. The incidence of GBS disease in non-pregnant adults has increased in recent years [3-5]. In adults, *S. agalactiae* may cause meningitis or septicaemia as well as localized infections such as subcutaneous abscesses, urinary tract infection or arthritis [3]. The drivers behind emergence of *S. agalactiae* disease in adults are poorly understood.

To study the epidemiology of *S. agalactiae*, numerous molecular methods have been used. This includes comparative typing methods, such as pulsed field gel electrophoresis (PFGE), which is suitable for outbreak investigations [6-8]. For population genetic analyses, highly standardized and portable typing methods are preferable, e.g. multilocus sequence typing (MLST), which targets the core genome, or 3-set genotyping, which targets the accessory genome content of *S. agalactiae*[9-11]. MLST is an important tool for molecular epidemiology because the MLST databases for individual pathogen species currently cover far more isolates than have been characterized based on whole genome sequencing [12]. Similarly, isolates that have been characterized by 3-set genotyping still outnumber isolates that have been characterized by whole genome sequencing, thus providing a less detailed but broader frame of reference than offered by whole genome sequences. MLST is an unambiguous method based on sequencing of the internal portion of selected housekeeping genes [13]. It is used to define sequence types (STs), which may be associated with specific disease syndromes. For example, ST17 is more prevalent among isolates from invasive disease in infants than among carriage isolates from pregnant adults [1,13]. Three-set genotyping encompasses molecular serotyping (MS) and profiling of surface protein genes and mobile genetic elements (MGE), and allows for further differentiation of isolates belonging to the same ST [11]. For example, ST283 isolates with molecular serotype III-4, C-α protein and C-α protein repeating units and the MGEs IS1381, ISSag1, and ISSag2 are associated with the emergence of GBS meningitis in adults in Southeast Asia [7,8].

Invasive disease due to *S. agalactiae* is not limited to humans. Other species affected include terrestrial mammals such as cattle, dogs and cats [14,15] and aquatic or semi-aquatic species such as sea mammals [16,17], crocodiles [6], bullfrogs [18] and fish [16,19]. Outbreaks of streptococcosis due to *S. agalactiae* have been described in wild fish, e.g. in mullet in Kuwait bay [16,20] and in giant Queensland Grouper and other wild fish in Australia [21]. *S. agalactiae* is also a major pathogen in farmed fish, particularly in tilapia [22-24]. Consumption of fish has been associated with an increased risk of *S. agalactiae* serotype Ia and Ib colonization in people [25]. Furthermore, MLST, molecular serotyping and challenge studies have shown that invasive disease in humans and fish may be caused by the same strains of *S. agalactiae*[16,19]. The aim of the current paper is to enhance our knowledge of the molecular epidemiology of *S. agalactiae* in fish and other aquatic species, with emphasis on use of standardized typing systems that cover housekeeping genes as well as virulence genes and that allow for assessment of transmission potential between aquatic species and humans based on comparison with existing databases.

## Methods

### Isolate collection and identification

**Figure 1:**
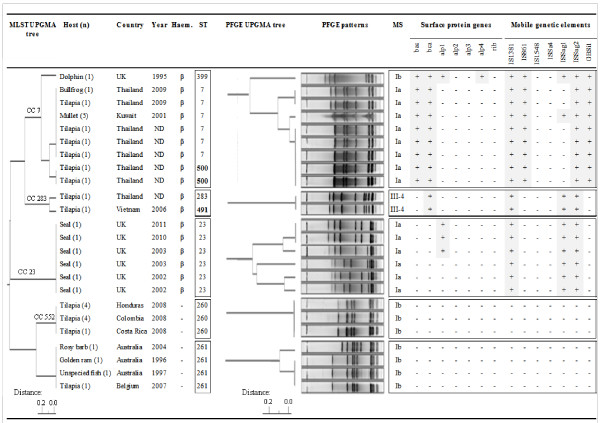
**Overview of *Streptococcus agalactiae *origin, isolate number (n) and results of phenotypic and genotypic characterization.** Results include analysis of haemolysis (Haem), multilocus sequence typing (MLST), pulsed-field gel electrophoresis (PFGE), molecular serotyping (MS), and profiling of surface protein genes and mobile genetic elements. Trees for MLST and PFGE results were constructed using unweighted pair group method analysis (UPGMA). Boxes enclose major clonal complexes (CCs) or sequence types (STs). STs shown in bold were first identified in the current study. ND: not determined.

A collection of 34 *S. agalactiae* isolates recovered from aquatic hosts was assembled, including isolates from poikilothermic and homeothermic host species originating from multiple countries and continents (Figure 1). Of 34 isolates, 13 represented 3 separate disease outbreaks (5 isolates from an outbreak Kuwait, 4 from Honduras and 4 from Colombia) with the remaining 21 isolates each representing a single, unrelated outbreak or death. Thus, isolates in this study represented 24 epidemiologically independent events. Most fish isolates (n=18) originated from infections in farmed tilapia (*Oreochromis* sp.) from Honduras, Colombia, Costa Rica, Belgium, Thailand and Vietnam. The remaining fish isolates originated from infections in wild Klunzinger’s mullets (n=5; *Liza klunzinger*) that were part of an outbreak of streptococcosis in Kuwait or from ornamental fish from Australia, namely a rosy barb (*Puntius conchonius*), a golden ram (*Mikrogeophagus ramirezi*) and an undetermined fish species. Sea mammal isolates (n=7) were recovered at post-mortem from lung swabs of 1 bottlenose dolphin (*Tursiops truncatus*) and 6 grey seals (*Halichoerus grypus*) that had stranded at various sites around the coast of Scotland. Finally, one amphibian isolate originating from an infected farmed bullfrog (*Rana rugurosa*) in Thailand was available for molecular characterisation.

Bacterial isolates were streaked onto 5% sheep blood agar plates (E&O Laboratories, Bonnybridge, United Kingdom) and grown aerobically at 27 to 28°C for 48h in order to assess purity and haemolysis properties. This temperature is commonly used for culture of *S. agalactiae* from fish [26]. Isolates were checked for Gram reaction and morphology and tested in a group B-specific latex agglutination test (Slidex Strepto Plus B; bioMérieux, Marcy L'Étoile, France). Single colonies were transferred to Brain Heart Infusion (BHI) broth (Oxoid, Basingstoke, United Kingdom) and incubated with gentle shaking at 28°C for 12h (ß-haemolytic strains, fast growing) or 48h (non-haemolytic strains, slow growing). Species identity of *S. agalactiae* was confirmed by polymerase chain reaction (PCR), using forward primer STRA-AgI (5^′^-AAGGAAACCTGCCATTTG-^′^3) and reverse primer STRA-AgII (5^′^-TTAACCTAGTTTCTTTAAAACTAGAA-3^′^), which target the 16S to 23S rRNA intergenic spacer region [27]. Broth cultures were also used for PFGE as described below.

### Comparative typing: PFGE

Bacterial cells were pelleted by centrifugation of 1 ml of incubated BHI, re-suspended in 0.5 ml of TE buffer (10 mM Tris-HCl, 1mM EDTA), warmed to 56°C and mixed with 0.5 ml of 2% (weight/vol) low-melting point agarose (Incert agarose; Lonza, Slough, United Kingdom) in TE buffer. The mixture was then pipetted into reusable plug moulds (Catalogue number 170-3622; BioRad Laboratories, Hemel Hempstead, United Kingdom) producing 20 × 9 × 1.2 mm^3^ agarose blocks. Each solidified plug was placed into 2 ml of TE buffer containing 4 mg of lysozyme (Sigma Aldrich, Poole, United Kingdom) (2 mg ml^-1^) and incubated overnight at 37°C with gentle shaking. The buffer was then replaced with 2 ml of ES buffer (0.5 M EDTA–1% (weight/vol) *N*-lauroyl sarcosine [pH 8.0 to 9.3]) supplemented with 4 mg of proteinase K (Promega, Southampton, United Kingdom) (2 mg ml^-1^) and incubated at 56°C for a minimum of 48 hr. Plugs were washed 6 times for 1 hr in TE buffer at room temperature and with gentle shaking. A slice (4 × 4 × 1.2 mm^3^) from each plug was exposed to digestion with restriction endonuclease *Sma*I (20 U in 100 μl of fresh reaction buffer; New England Biolabs, Hitchin, United Kingdom) at 25°C overnight. PFGE was performed with a CHEF-mapper system (BioRad Laboratories) in 0.5 × TBE using a 1% (weight/vol) agarose gel (Pulsed Field Certified Agarose, BioRad Laboratories), a run time of 24 hr and switch time of 3-55 s (linear ramp) at 14°C. Patterns were observed by UV transillumination after SYBR Gold staining (Invitrogen, Paisley, United Kingdom). Computer-assisted data analysis and dendogram construction were performed with Phoretix 1D Pro software (TotalLab Ltd, Newcastle upon Tyne, United Kingdom). Similarities between PFGE patterns were also assessed visually using standard criteria [10].

### Housekeeping genes: multilocus sequence typing

MLST consisted of the amplification by PCR and sequencing of seven housekeeping genes, namely *adhP*, *atr*, *glcK*, *glnA*, *pheS*, *sdhA*, and *tkt*[13]. Consensus sequences were trimmed in SeqMan (DNAStar, London, United Kingdom), and the *S. agalactiae* database [28] was used for allele and sequence type (ST) assignments. Sequences of novel alleles were submitted to the database curator for allocation of new allele numbers and STs; these are now available in the database. The unweighted pair group method in PHYILIP and Phylodendron was used to visualize the relationship between allelic profiles obtained from the isolates. The complete allelic profile list from the *S. agalactiae* MLST database was downloaded (last accessed 7 November 2012) [28] and eBURST groups were identified based on sharing of 6 out of 7 alleles using standard eBURST methodology [29]. In addition, a population snapshot of the entire *S. agalactiae* population was created in eBURST to show the position of STs from our study in relation to all known STs, which predominantly originate from isolates of human origin. Finally, for STs that were identified in the current study and that did not form part of an eBURST group, the existence of double locus variants (DLVs) and triple locus variants (TLVs) was explored via ST query in the *S. agalactiae* MLST database [28].

### Virulence genes: three-set genotyping

A 3-set genotyping system, comprising MS, surface protein gene profiles and MGE profiles, was used. Molecular serotyping was performed using multiplex-PCR assays [16]. Non-typeable (NT) isolates were further investigated using other primer sets [30] and serosubtyping of MS III isolates was performed [31]. Presence of surface protein genes was determined by PCR and sequencing of PCR products, using primers targeting the *bca*, *bac*, *alp1*, *alp2*, *alp3* and *alp4* genes [32]. Finally, the prevalence of 7 MGE, corresponding to 1 group II intron (GBSi1) and 6 insertion sequences (IS1381, IS861, IS1548, ISSa4, ISSag1 and ISSag2) was evaluated by PCR and amplicon identity was confirmed by sequencing of PCR products [23,33].

## Results

### Isolate collection and identification

All isolates were Lancefield Group B, Gram-positive cocci appearing in pairs and chains. They were either β-haemolytic or non-haemolytic on sheep blood agar (Figure 1). All were confirmed as *S. agalactiae* by species-specific PCR.

### PFGE analysis

All isolates were typeable by *Sma*I macrorestriction and 13 pulsotypes were identified. Pulsotypes were indistinguishable when multiple isolates from a single outbreak were analysed. In some cases, pulsotypes were also indistinguishable for isolates from different host species or countries, e.g*.* for bullfrog and tilapia isolates from Thailand or for tilapia isolates from Honduras, Colombia and Costa Rica (Figure 1). Despite efforts to identify potential epidemiological relationships between farms sharing the same pulsotype, e.g. through shared broodstock or feed companies, no such links could be identified and each outbreak is considered to be epidemiologically independent.

### MLST and eBURST analysis

Among the 34 *S. agalactiae* isolates, 8 STs were observed, including 2 new STs, i.e. ST491 and ST500 (Figure 1). The new STs were SLVs of known STs and resulted from single nucleotide changes. STs were identical when multiple isolates from a single outbreak were analysed. In several cases, STs were also identical between isolates from epidemiologically unrelated animal deaths, outbreaks, countries or host species. For example, ST261 was associated with three epidemiologically unrelated fish death in Australia; ST260 was found in tilapia from Honduras, Colombia and Costa Rica; and ST7 was found in a bullfrog and tilapia from Thailand and in mullet from Kuwait (Figure 1). The ST7 isolates from Thailand originated from 5 provinces (Nakhon Sawan Province – frog farm; Kanchanaburi Province – tilapia farm; Nakhon Pathom Province - 2 tilapia farms; and Saraburi Province – tilapia farm) and were epidemiologically unrelated. The two ST500 isolates from Thailand originated from tilapia farms in Mukdahan Province and Phetchaburi Province, respectively.

**Figure 2:**
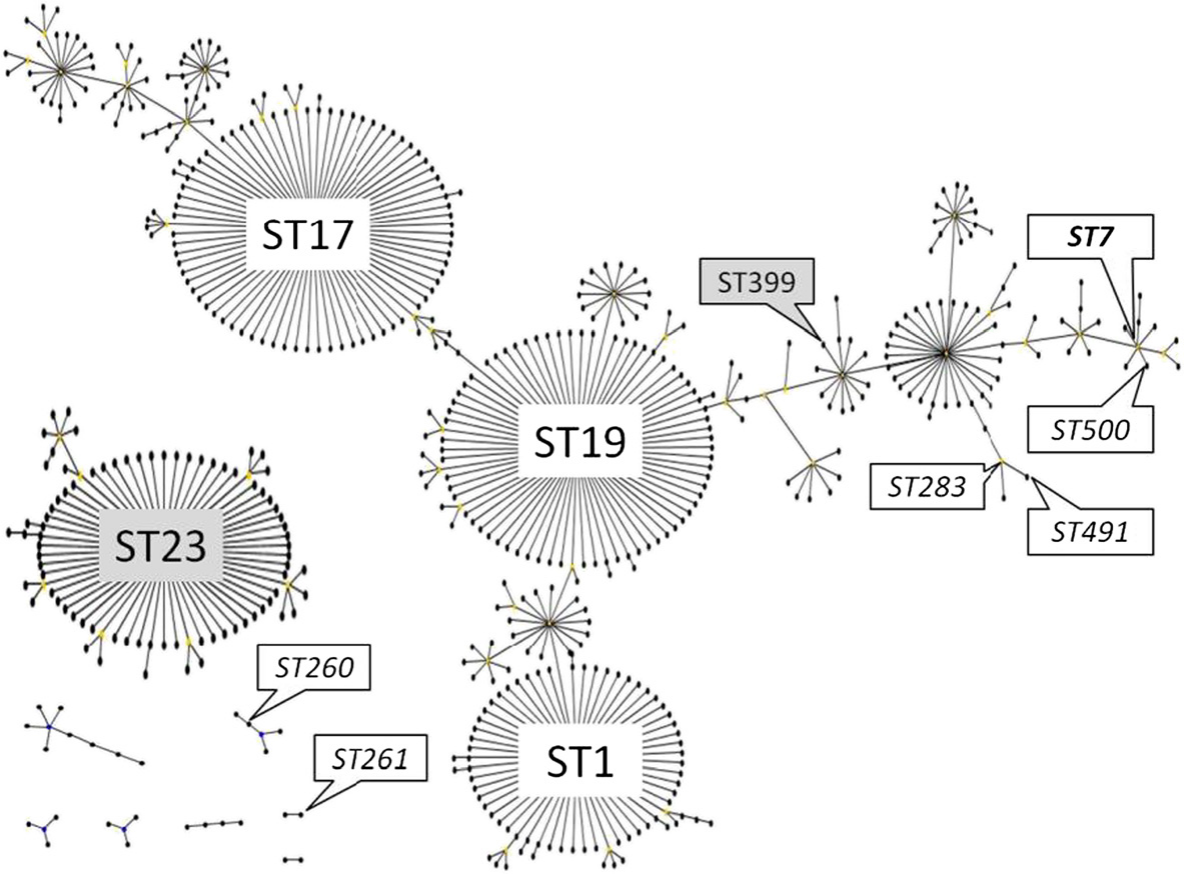
**Population snapshot of *S. agalactiae *constructed in eBURST.** In addition to the 9 eBURST groups that are shown, 36 singletons were present in the database (last accessed 7 November 2012). Founders of major clonal complexes (ST1, ST17, ST19, all of which form part of eBURST group 1, and ST23, which is the founder of eBURST group 2) and sequence types (ST) identified in the current study are labelled. Italics indicate STs isolated from fish, bold italics indicate the ST from fish and a frog, and shaded labels indicate STs from sea mammals. All β-haemolytic *S. agalactiae* isolates from fish belonged to a single branch of eBURST group 1, all seal isolates (n=6) belonged to eBURST group 2 and all non-haemolytic isolates belonged to two small eBURST groups that included ST260 and ST261.

**Figure 3:**
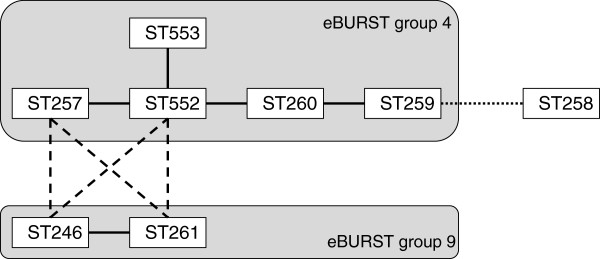
**Overview of sequence types (ST) of *Streptococcus agalactiae *that have only been isolated from poikilothermic host species (fish, frog).** STs that share 6 of 7 alleles, i.e. single locus variants, are connected by full lines and grouped into eBURST groups. STs that are members of different eBURST groups but share 5 of 7 alleles, i.e. dual locus variants, are connected by dashed lines. ST258 shares 4 of 7 alleles with ST259 and the relationship of this triple locus variant to the eBURST groups is represented by a dotted line. All STs in this diagram share fewer than 4 alleles with all STs that have been identified in homeothermic host species (e.g. humans and seals).

E-burst analysis (Figure 2) showed that all piscine isolates from Asia and the Middle-East and the frog isolate from Asia (ST7 and its SLV ST500; ST283 and its SLV ST491) belonged to 2 related subgroups, both of which are part of eBURST group 1. The bottlenose dolphin isolate from the UK (ST399) also belonged to eBURST group 1. This large eBURST group includes a number of major subgroups that used to be separate eBURST groups or clonal complexes (CCs). For ease of reference and comparison with the literature, such subgroups or subCCs are indicated in the figure and subsequent text by their founding ST. All grey seal isolates from the UK belonged to ST23, which is the founder of eBURST group 2 or CC23 and not related to ST7 or ST283. Piscine isolates from Latin America (ST260) were part of a small eBURST group that also includes ST257, ST259, ST552 and ST553 (Figure 2). The most likely founder of this eBURST group is ST552 and the group is also referred to as CC552. Based on additional analysis of DLVs, ST261 and ST246 may also be related to CC552 whilst ST258 is a TLV of CC552 (Figure 3).

### Three-set genotyping

Using the method of Evans and colleagues [16], isolates were identified as serotype Ia, Ib or NT. Further investigation of NT isolates with additional primer sets [30,31] showed that the isolates belonged to serotype III subserotype 4. Based on the combination of serotype, surface protein genes and MGE, seven 3-set genotypes were distinguished (Figure 1). Three-set genotypes were identical when multiple isolates from a single outbreak were analysed. Piscine and amphibian isolates from Asia and the Middle-East and all mammalian isolates were positive for IS1381 and ISSag2. IS861 was always found in combination with GBSiI and *vice versa* but rarely in combination with ISSag1. ISSag1 was found in all mammalian isolates tested but only 3 of 21 epidemiologically independent non-mammalian isolates carried ISSag1. When the Cβ protein gene (*bac*) was present, it was always found in association with the Cα protein gene (*bca*) but *bca* could also present in the absence of *bac* (Figure 1). Piscine isolates from Latin America (n=6), Australia (n=3) and Europe (n=1), all shared serotype Ib (Figure 1) but none of the surface protein genes or MGE investigated in this study were detected in any of these isolates.

### Comparison across methods

All β-haemolytic isolates (n=21, representing 17 epidemiologically independent events) belonged to CCs that are also found in humans and carried at least 3 MGEs (Figure 1). Each CC correlated with a PFGE cluster, although MLST could be more discriminatory than PFGE and vice versa. For example, multiple PFGE types were identified in ST7 and in ST23 (Figure 1). Conversely, multiple STs were identified within PFGE types in CC7 (ST7 and ST500) and CC283 (ST283 and ST491). Results from 3-set genotyping were concordant with MLST and PFGE typing and origin of isolates. All isolates from CC7 (n=14, representing 9 epidemiologically independent events) carried at least 2 surface protein genes and 4 MGEs (IS1381, IS861, ISSag2 and GBSi1), which is more than was observed in any other CC in this study. Within CC7, the dolphin isolate was the most divergent isolate based on MLST, PFGE typing, serotyping and number of surface protein genes. The dolphin isolate and the outbreak strain from Kuwait had one extra MGE, ISSag1, compared with isolates from Thailand (Figure 1), which were identical to each other in 3-set genotype. Isolates in CC283 (n=2) and CC23 (n=6) carried the same MGEs (IS1381, IsSag1 and IsSag2) but differed from each other in presence of surface protein genes. Within CC23, two closely related PFGE clusters were observed that corresponded with presence or absence of the surface protein gene alp1. All non-haemolytic isolates (n=13, representing 7 epidemiologically independent events) belonged to STs that have not been identified in humans and none of these isolates carried any of the surface protein genes or MGEs that were examined (Figure 1).

## Discussion

*Streptococcus agalactiae* from sea mammals, fish and a frog belonged to 4 subpopulations based on a combination of two standardized typing methods which target the core genome and the accessory genome, respectively. Of the 4 subpopulations that were identified, 3 have also been found in humans, both as carriage strains and as the cause of invasive disease in neonates or adults, whilst to date the fourth one has only been reported from poikilothermic animals.

### *S. agalactiae* CC283 is associated with invasive disease in humans and fish

ST283 with molecular serotype III-4 has been associated with invasive disease in non-pregnant adults in Hong Kong [7] and was isolated from fish in Thailand in our study. Isolates from humans and fish also shared the presence of the C-alpha encoding gene as well as identical MGE profiles. The same 3-set genotype was found in an SLV of ST283, the novel ST491, which was isolated from fish in Vietnam in our study. ST283 and another one of its SLVs, ST11, have previously been linked to an increase in group B streptococcal meningitis in adults in Southeast Asia [7]. In France, ST283 serotype III has been isolated from cases of osteoarticular disease in non-pregnant adults [34]. The 3-set genotype shared by human isolates from Hong Kong and tilapia isolates from Southeast Asia in our study had already been reported from tilapia in Thailand, but MLST data were not published for those isolates [23]. The recent emergence or recognition of invasive ST283 and its SLVs in humans and fish in Southeast Asia suggests that there may be an epidemiological connection between the two host species, as previously described for a closely related streptococcal species, *Streptococcus iniae*[35]. Such a connection could result from human-to-animal transmission, animal-to-human transmission or joint exposure to a shared source. Further study of ST283 and the epidemiological connection between humans and fish will be needed to elucidate potential transmission mechanisms and risks.

### *S. agalactiae* CC7 is associated with carriage and disease in humans, a bullfrog, fish and dolphins

In humans, ST7 causes invasive disease in neonates and adults [13,16] and the pathogenicity of human ST7 isolates to fish is well-established [19]. ST7 may also occur as vaginal carriage isolates in humans [13,36]. ST7 was held responsible for a major fish kill in Kuwait bay [16] and results from our study using isolates from different fish from the same outbreak confirm this. Human sewage was considered a likely source of this outbreak [20], a route of transmission that is consistent with commensal occurrence of strains in the human urogenital tract. We report here for the first time the detection of ST7 in an amphibian. Previous reports on the occurrence of *S. agalactiae* in frogs mention non-haemolytic GBS strains [18,37] but all ST7 isolates in our study and in previous studies of aquatic *S. agalactia*e were β-haemolytic. Thus, it is unlikely that infections described previously in frogs were due to ST7. Like most ST7 isolates in our study, the frog isolate originated from Thailand, where this ST is common in farmed tilapia (Figure 1).

*S. agalactiae* has been isolated from captive and wild dolphins [17,38]. ST7 was cultured from a bottlenose dolphin (*Tursiops truncates)* that died during the Kuwait Bay fish kill but no definitive link between bacterial isolation and death could be established [38]. Similarly, we describe the first case of ST399 in a free-ranging bottlenose dolphin calf from Scotland without definitive evidence of a causal association with the animal’s death, which was attributed to trauma and infanticide. ST399 is a rare SLV of ST12 and does not appear to be closely related to ST7 in eBURST analysis of the current MLST database (Figure 2). However, ST399 is a DLV of ST7 and alternative methods, e.g. clustering of MLST data using the unweighted pair group method, suggest that ST399 should be classified as a member of CC7 [39]. Due to the low number of dolphin isolates available, it is not possible to determine whether the isolation of two CC7 strains from temporally and geographically unrelated dolphins is coincidental or reflective of a host predilection. Like ST7, ST399 may occur as a vaginal coloniser in healthy women [39]. Thus, its presence in sea water could result from microbial contamination by human effluent.

### *S. agalactiae* ST23 is associated with humans and seals but not with fish

*Streptococcus agalactiae* has been detected in grey seals (*Hallichoerus grypus*) and in Antarctic fur seals (*Arctocephalus gazelles*) but those descriptions predate the development of MLST [40,41]. *S. agalactiae* was identified in 9 grey seals under the Scottish Strandings Scheme whereas examination of a larger number of common seals (*Phoca vitulina*) under the same Scheme failed to recover *S. agalactiae*, suggesting that among Scottish pinnipeds, *S. agalactiae* has a preference for grey seals. Complete molecular typing data was available for 6 isolates, which are included in the current study, whilst MLST data was available for the remaining 3 isolates. One of the grey seals had died of a systemic infectious process, whilst other animals with *S. agalactiae* died with signs of storm damage, hypothermia, starvation, trauma or lung emphysema, in agreement with previous studies [40,41]. All seal isolates (n = 9) belonged to ST23. Within ST23, molecular serotypes Ia and III predominate [1,14]. ST23 serotype Ia is linked to humans whereas ST23 serotype III is primarily found in dairy cattle where it is a causative agent of mastitis, inflammation of the mammary gland [14,15]. All seal isolates included in the current study (n = 6) had serotype Ia, suggesting a human origin. In humans, ST23 is common as vaginal-rectal carrier strain in adults although it may also cause neonatal invasive disease [1,13]. Given the predominant niche of ST23 in humans, it is conceivable that its presence in seals is due to microbial contamination of surface water. ST23 probably has the broadest known host range of all *S. agalactiae* STs. Both homeothermic and poikilothermic species can be affected, including humans, cattle, dogs, crocodiles and seals [6,14,15]. Despite the high prevalence of ST23 in humans, its wide host range and its ability to affect aquatic mammals and semi-aquatic reptiles, there are no reports on occurrence of ST23 in fish. This may reflect the relatively small number of fish isolates characterized to date or it may indicate true biological differences, e.g. an inability to infect fish. Challenge studies using ST23 are required to assess its ability to cause disease in fish. If ST-associated differences in virulence are confirmed, comparative genomic analysis of human, fish, seal and bovine isolates may help to identify molecular correlates of virulence.

### *S. agalactiae* ST260 and ST261 are associated with fish but not with humans

The final subpopulation in our collection consisted of non-haemolytic strains of *S. agalactiae*. Non-haemolytic *S. agalactiae* may cause invasive disease such as endocarditis in adult humans [42] but no MLST data on non-haemolytic human isolates could be found. The prevalence of non-haemolytic *S. agalactiae* among carriage isolates has been estimated at 5 to 8%, although this value may be underestimated in studies that use β-haemolyis as a diagnostic criterion for identification of the organism [43]. All non-haemolytic isolates in our collection belonged to serotype Ib, a serotype that has been associated with β-haemolytic and non-haemolytic human isolates [1,37]. The subpopulation of non-haemolytic serotype Ib isolates in our study encompassed all fish isolates that did not originate from Southeast Asia, suggesting an association between geographic origin and strain. The association with host species and geographic origin is not absolute, as β-haemolytic serotype Ib isolates and ST261 have also been reported from frogs [37,44] and ST261 has been reported in fish from Indonesia [45]. This is the first report of ST261 in aquarium fish, which originated from Australia. Outbreaks of streptococcosis in wild fish have occurred repeatedly in Australia in the past few years [21]. The isolates causing disease in Queensland grouper and other reef fish were non-haemolytic with serotype Ib, suggesting that they belong to the fish-associated subpopulation of *S. agalactiae*. Human strains, such as ST7 or ST23, could come into contact with reef fish through surface water contamination by effluent or divers but would be expected to be haemolytic. It is unknown whether there is an epidemiological connection between disease in aquarium fish and reef fish, e.g. due to capture of reef fish or release of aquarium fish into the wild.

Using standard eBURST group definitions, ST260 but not ST261 is recognized as part of CC552 (Figure 2). However, ST261 is a DLV of multiple CC552 members and could be considered a member of the same group (Figure 3). This group also includes ST246, which has been isolated from trout, and ST257 and 259, which have been isolated from tilapia [14,16]. ST258, which has been isolated from striped bass [16], is loosely connected to this group, which does not include any isolates from homeothermic host species. Using the 3-set genotyping system, no surface protein genes or MGEs were detected among isolates from this group, further supporting that it is not closely related to any of the known clonal complexes of *S. agalactiae* found in humans. ST261 was recently discovered in doctor fish (*Gara rufa*) that are used in foot spas to remove dead skin from people’s feet and concern has been expressed that repeated exposure of fish-adapted strains to such an environment could eventually lead to human infections [45]. In the laboratory, members of the group that includes ST260 and ST261 do not grow well at 37°C, which may explain their current absence from homeothermic species. A vaccine to protect fish from non-haemolytic *S. agalactiae* is commercially available, but this vaccine does not provide protection to haemolytic strains [14]. Thus, vaccination of fish can be used to limit production losses in some situations, but it does not protect against the most commons strains in Southeast Asia or against zoonotic infections from fish or fish products.

## Conclusions

Based on standardized molecular typing of housekeeping genes and virulence genes, *S. agalactiae* strains that have previously been associated with asymptomatic carriage and adult invasive disease in humans can also be found in fish, frogs and sea mammals. In particular, strains belonging to ST23, which is a common carriage strain in humans, were associated with seals, where they may be indicators of environmental pollution rather than causative agents of disease. ST23 was not identified in any fish. Strains belonging to ST7 were associated with a bullfrog and fish from South-East Asia whilst strains belonging to ST283 and showing the same virulence gene profile as human invasive isolates were isolated from fish in Asia. This suggests that there may be exposure of humans and fish to similar environmental sources of ST7 and ST283, or transmission of *S. agalactiae* between the different host species. Finally, strains belonging to ST260 and ST261 were associated with fish from the Americas, Europe and Australia. These strains, and other members of their clonal complex, have only been reported from poikilotherms. Comparative genomic analysis and functional studies of strains that do not appear to affect fish, e.g. ST23, and strains that primarily affect fish, e.g. ST260 and ST261, may provide insight into host-adaptation of *S. agalactiae*. Epidemiological studies are needed to provide insight into the likelihood and routes of interspecies transmission of strains that are associated with fish, sea mammals and invasive disease in humans as well as control measures needed to prevent transmission and disease.

## Abbreviations

CC: Clonal complex; DLV: Dual locus variant; GBS: Group B streptococcus; MGE: Mobile genetic element; MLST: Multilocus sequence typing; MS: Molecular serotype; NT: Non-typeable; PFGE: Pulsed field gel electrophoresis; SLV: Single locus variant; ST: Sequence type; TLV: Triple locus variant.

## Competing interests

The authors declare that they have no competing interests.

## Authors’ contributions

CD participated in study design and acquisition of isolates, conducted the laboratory work on piscine and amphibian isolates and collated and analysed the data; MC, JT and MF participated in conception and design of the study and MC contributed to acquisition of isolates from fish and amphibians; JP conducted laboratory work on sea mammal isolates; GF and MD conducted field and laboratory work resulting in isolation of sea mammal isolates; RZ conceived of the study and participated in its design and coordination. CD and RZ drafted the manuscript. All authors contributed to, read, criticized and approved the final manuscript.
